# Effect of Sodium Chloride on the Surface and Wetting Properties of Aqueous Solutions of Cocamidopropyl Betaine

**DOI:** 10.1007/s11743-014-1644-8

**Published:** 2014-09-26

**Authors:** Katarzyna Staszak, Daria Wieczorek, Katarzyna Michocka

**Affiliations:** 1 Institute of Technology and Chemical Engineering, Poznań University of Technology, pl. Skłodowskiej-Curie 2, 60-965 Poznań, Poland; 2 Department of Technology and Instrumental Analysis, Faculty of Commodity Science, Poznań University of Economics, al. Niepodległości 10, 61-875 Poznań, Poland

**Keywords:** Cocamidopropyl betaine, Amphoteric surfactant, Surface properties

## Abstract

Surfactants are important ingredients of personal care products and household products. The main characteristic of these compounds is to decrease the surface tension of solvent and resulting many properties such as contact angle, foam properties etc. The coexistence of other ingredients in the product may affect the properties of surfactants. One of the main components contained in almost every personal care and household product is sodium chloride. The main aim of this work was to determine the effect of this salt on some surface and usage properties of cocamidopropyl betaine (CAPB). From our experiments it was shown that the effect of added sodium chloride in the aqueous solutions of CAPB on the properties is the opposite to the one described in the literature for cationic and anionic surfactants, i.e., CMC increases with increasing ionic strength, foam height decreases with increasing salt concentration. Our investigation showed that sodium chloride makes worse the properties of the CAPB solutions examined in this work.

## Introduction

Surfactants are one of the most important groups of organic chemicals, and are used in vast amounts in domestic and industrial applications [1, 2]. They are designed to remove dirt, sweat, sebum, and oils from the skin and other surfaces [3]. One of the classes of surface active agents is zwitterionic surfactants. These surfactants exhibit excellent surface properties, i.e., low surface tension and a critical micelle concentration. Zwitterionic surfactants are good wetting agents and can be high, moderate or low foaming surfactants [4].

**Fig. 1:**
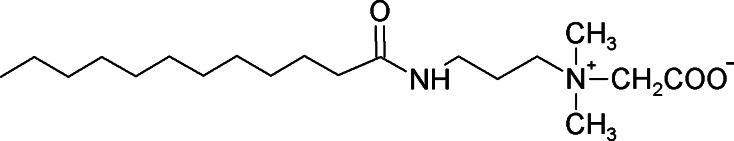
General formula of cocobetaine

Probably the most important class of amphoteric surfactants is amidopropyl betaines, in particular Cocamidopropyl betaine (CAPB) [5]. It is a popular and widely used zwitterionic surfactant. Cocamidopropyl betaine is predominately used as a cosmetic ingredient and as a detergent (50 % of the produced volume in Europe—29,500 tons/year) [6]. It is commonly used in cosmetics and household chemicals [7]. For example it is used in cosmetics and personal hygiene products, mainly in rinse-off products such as shampoos, but also in roll-on deodorants, contact lens solutions, toothpaste, makeup removers, bath gels, skincare products, cleansers, liquid soaps, anti-dandruff products, and exfoliating and peel-off products, in the preparations for the treatment of acne and conditioners, as well as eye make-up products and antiseptics [7–13]. Moreover it is use as a detergent includes hand washing agents, and hand dish washing agents [6]. Due to the mild nature and low degree of skin irritation it is also used in cosmetics for children. Cocobetaine is also present with other surfactants (anionic, nonionic) in commercially available products. This combination is designed to change the surface properties. An example of such a combination is a mixture of cocobetaine and sodium lauryl sulfate (SLS) as the primary surfactant, which reduces the irritation of the skin and mucous membranes, strengthens the feeling on the skin, and increases the organoleptic sensations [5, 10, 12, 14, 15]. Cocobetaine is a light yellow and clear liquid with a gentle odor. Its density at 25 °C is 1.043 g/cm^3^. It is easily soluble in water and its 10 % solution has an acidic reaction with a pH of about 5–6 [16]. The general formula of cocobetaine is shown in Fig. 1 [16].

A literature survey shows that chaotropic ions influence some properties like solubilization, micellization and rheology. The addition of salts to aqueous surfactant solutions may result in a modification of both intramicellar and intermicellar interactions [17]. Accordingly, a fundamental understanding of how salts affect the behavior of aqueous surfactant solutions may lead to a more effective utilization of this material in various practical applications. Studies on the effect of sodium chloride on the micellar properties are mainly focused on the cationic and anionic surfactants [18–22].

### Fundamentals

Sodium chloride is used as a thickener in shampoos and conditioners containing sodium lauryl sulfate. It is a contributing factor to the eye irritation experienced with most shampoos, and it may also cause dry and itchy scalp. Salt may also cause some hair loss [23]. The addition of salts to the aqueous solution of the surfactants changes the solution properties, such as the critical micellar concentration, as well as the phase behavior of the surfactant solution, as well as the critical micelle concentration [17]. Because salts are added to the personal care products, it is important to assess the impact of salt on the behavior of such solutions.

The aim of this study was to check the effect of sodium chloride on the surface and wetting properties of aqueous solutions of CAPB.

In the presence of sodium chloride, cocamidopropyl betaine may be described as each the inner salt or as the respective sodium salt [6].

## Experimental

The sample of cocobetaine was purchased from one of the cosmetics companies making it available on the Polish market. It was stored in a refrigerator and not used after the expiration date specified by the producer. According to the manufacturer's information, the commercial CAPB consists of 30 % cocamidopropyl betaine and 70 % water, without sodium chloride [24].

The samples of cocobetaine used to measure surface tension were prepared as aqueous and salt solutions (NaCl, from POCH S.A, Poland) of surfactant. The initial concentration of cocobetaine solution was 10 g/dm^3^. The surface tension measurement was made for stock aqueous and salt solutions and for the following 18 solutions obtained by a serial dilutions method. The measurement was carried out for aqueous and sodium chloride solutions with four concentrations: 0.01, 0.05, 0.2 and 2 M using a pendent drop tensiometer TRACKER (I.T. Concept, France). The tensiometer captures the image of an air drop immersed in the aqueous solution of surfactant and records the drop shape as a function of time. The drop shape is determined by the surface tension of the liquid, gravity, and the density difference between the air and surrounding medium. The drop image is analysed with a profile fitting method in order to determine the contact angle and the surface tension. Measurements of surface tension were performed at constant temperature for aqueous solutions at 297 K. Measurement duration depends on concentration of surfactants in the sample and was conducted to reach equilibrium. Additional physicochemical analyses on the basis of the surface tension data were carried out using the Szyszkowski and Frumkin equation [25]. Contact angle measurements were performed with the same equipment for water or aqueous salt solutions at a concentration of cocobetaine 1, 0.01, 0.0001 g/dm^3^. Surfaces used for measurements of contact angle were: aluminium, paraffin, PVC (poly(1-chloroethylene)), PTFE (poly(1,1,2,2-tetrafluoroethylene)), glass, PE (polyethene), PMMA (poly(methyl 2-methylpropenoate)).

The foaming properties of the water and aqueous salt solutions of cocobetaine were investigated in a glass apparatus using the Ross-Miles method in accordance with the standard ASTM00A51E47 [26]. After a creating column of foam, the foam height and volume were measured as a function of time. Foam volumes recorded after 10 min were the basis for determining the stability of the produced foam. The measurements of foaming properties were made for water and aqueous salt solutions for six different concentrations of cocobetaine.

## Results and Discussion

During the measurement of the surface tension by the drop shape method, the dependence of surface tension *versus* time was observed. Because the adsorption process of surfactants is dynamic, exact equilibrium values of the surface tension are necessary to determine the adsorption isotherms. For surfactants with low surface activity the adsorption reaches its equilibrium in time on the order of seconds to hours. It was found that the time to reach the adsorption equilibrium decreases with the increase in the concentration of cocobetaine in the aqueous solutions and changed from about 600 to 2,400 s at the lowest measured concentration.

**Fig. 2:**
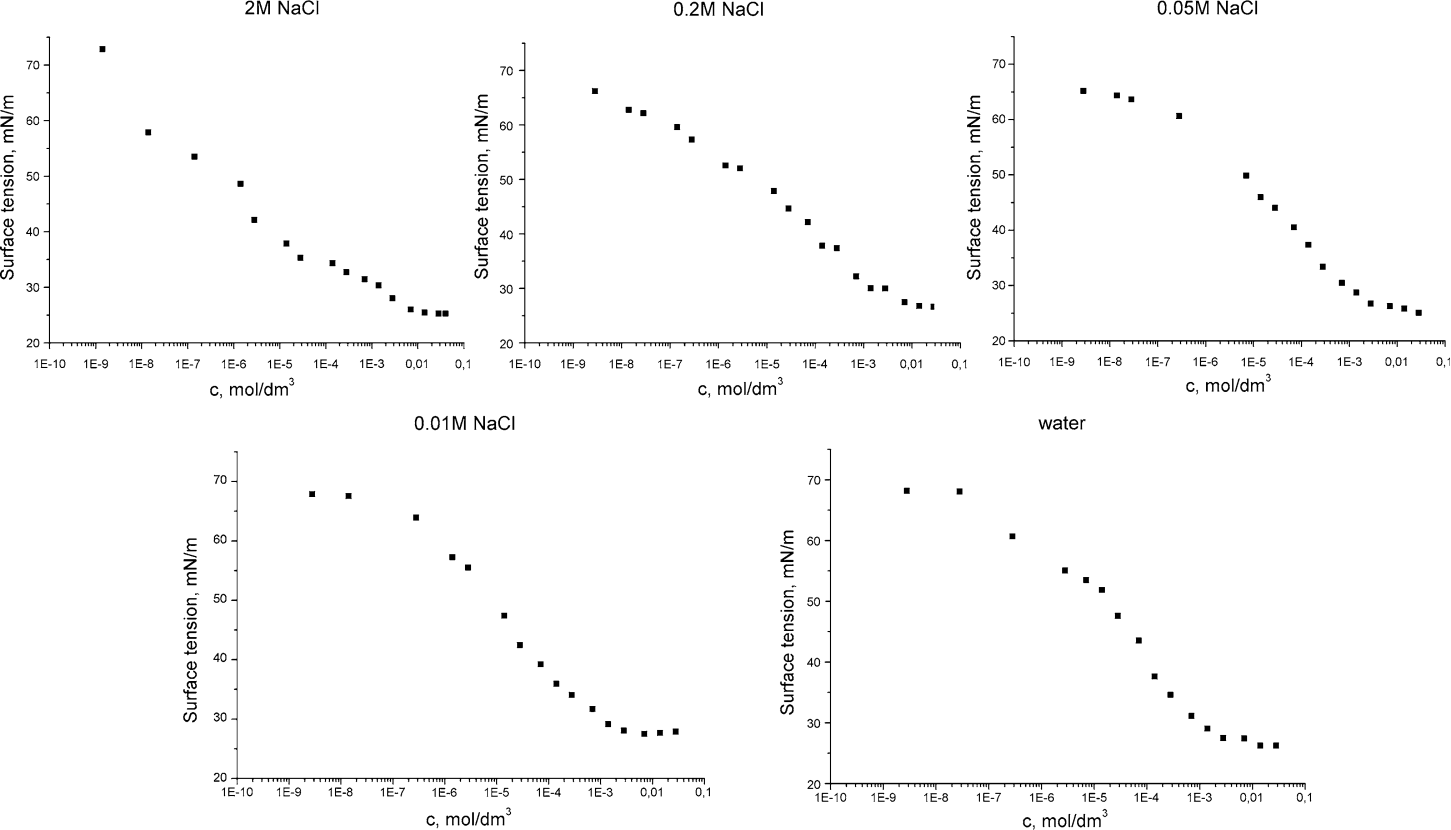
Surface tension isotherms for aqueous and salt solutions of cocobetaine

In Fig. 2, the plots of the surface tension *versus* the log of concentration are presented. It was found that cocobetaine lowered the surface tension in all system investigated. The minimum values of the surface tension were similar (about 25 mN/m) regardless of the sodium chloride concentrations. From these curves critical micellar concentration (CMC) values were estimated and are listed in Table 1. They were calculated for a molar mass of 356 which corresponds to the derivative of cocobetaine with 12 carbon atoms in the alkyl chain. As seen in Fig. 2 and Table 1, values of the CMC increase with increasing concentrations of salt in the aqueous solutions. The presence of the electrolyte generally causes a decrease in the CMC of most surfactants. The greatest effect is found for ionic materials. Addition of electrolytes decreases the repulsion between similarly charged ionic head groups within a micelle and therefore, the detergent monomers can pack tightly and the CMC is reduced. Non-ionic and zwitterionic surfactants display a much smaller effect or even reverse. The change in the CMC of these types of surfactants on the addition of electrolyte has been attributed mainly to the “salting out” or “salting in” of the hydrophobic groups in the aqueous solvent by the electrolyte, rather than to the effect of the latter on the hydrophilic groups of the surfactant [27].

**Table 1:** Adsorption parameters for cocobetaine in water/air systems

Adsorption parameter	Unit	2 M NaCl	0.2 M NaCl	0.05 M NaCl	0.01 M NaCl	Water
CMC	mol/dm^3^	7.02 × 10^−3^	5.60 × 10^−3^	2.23 × 10^−3^	0.89 × 10^−3^	0.28 × 10^−3^
*A*_Sz_	mol/dm^3^	1.16 × 10^−10^	1.24 × 10^−9^	3.16 × 10^−9^	2.79 × 10^−8^	4.65 × 10^−8^
*B*_Sz _× 10^2^	–	3.70	3.96	4.51	5.56	5.67
K or *S*z	–	0.977	0.991	0.988	0.993	0.990
*A*_F_	mol/dm^3^	3.93 × 10^−10^	3.85 × 10^−8^	1.11 × 10^−7^	7.95 × 10^−8^	1.96 × 10^−7^
*B*_F _× 10^2^	–	2.13	5.83	6.85	6.43	7.06
*A*′	–	2.23 × 10^−1^	2.73 × 10^−3^	0	−1.58 × 10^−3^	−7.59 × 10^−3^
K or F,	–	0.987	0.996	0.999	0.995	0.993
Γ^∞ ^× 10^6^	mol/m^2^	1.10	1.17	1.33	1.64	1.66
*A*_min_	nm^2^	1.50	1.42	1.25	1.01	0.998
Δ*G*_ads_	kJ/mol	−55.9	−50.2	−47.9	−42.5	−41.3

As was mentioned above, in the presence of sodium chloride in an aqueous solution of cocamidopropyl betaine could be described as the inner salt or as the respective sodium salt, and that could be a reason for the different behavior of this compound in systems with NaCl compared to systems with water. In the commercial products, salts are often added, and this should be taken into account to explain the different surface properties of cocamidopropyl betaine.

The measured change of the surface tension with concentration was fitted with the Szyszkowski isotherm:

1 $$\gamma^{\text{Sz}} = \gamma_{0} \left[ {1 - B_{\text{Sz}} \ln \left( {\frac{c}{{A_{\text{Sz}} }} + 1} \right)} \right]$$

where *γ*_0_ is the interfacial tension for concentration *c* = 0 and *A*_Sz_ and *B*_Sz_ are the adsorption coefficients. Using the Szyszkowski adsorption coefficients *A*_Sz_, *B*_Sz_, the surface excess at the saturated interface (Γ^∞^), the minimum molecular area in the adsorption layer at the saturated interface (*A*_min_) and the Gibbs free energy of adsorption (Δ*G*_ads_) could be estimated [28]. On the other hand, the Szyszkowski isotherm does not take into consideration the mutual interactions between the adsorbed molecules, therefore, the Frumkin isotherm might be more suitable for describing the experimental data as it does introduce such interaction. The Frumkin isotherm is represented by Eq. (2) in which *A*′ characterizes the mutual interactions between the adsorbed molecules [29]:

2 $$\gamma^{\text{F}} = \gamma_{0} \left[ {1 - B_{\text{F}} \ln \left( {\frac{c}{{A_{\text{F}} }} + 1} \right) - A'\left( {\frac{c}{{c + A_{\text{F}} }}} \right)^{2} } \right]$$

The surface tension measurements showed that the cocobetaine is surface active in all aqueous solutions and depends on the salts concentration. The results obtained, presented in Table 1 indicate that in the case of using water as the solvent, the surface concentration at the saturated interface Γ^∞^ is higher in comparison to the solution with various concentration of NaCl. In consequence, the statistical area occupied by the adsorbed molecules of cocobetaine at the saturated air/water interface is smaller in comparison to the system with salt. The same relationship was described for other zwitterionic surfactants: *n*-alkyl phosphocholines [30]. Salt addition (0.1 M NaCl) has resulted in a slight increase in the limiting area per molecule. Moreover the same effect was described for tripolar zwitterionic surfactants [31]. The results obtained suggest that the structures of the adsorbed monolayers formed at the interfaces considered are quite different. The molecules of the cocobetaine are much closer together at the air/water interface than in the case of the air/salt solution one. The different populations at the interface with the salt and the non-salt aqueous solution suggests a different orientation of the adsorbed molecules at the interfacial monolayer.

As indicated by the data presented in Table 1, the magnitude of Δ*G*_ads_ is also affected by the type of aqueous solution. The values of Δ*G*_ads_ calculated in systems with NaCl are more negative in comparison to the system without salt, and decrease with increasing salt concentration. Thus a less pronounced tendency for adsorption was observed in the systems with water. In other words, the results obtained suggest more spontaneous adsorption processes of molecules of cocobetaine in systems with NaCl in aqueous solutions.

The analysis of the mutual interactions between the adsorbed molecules on the ground of the magnitude of *A*′ values indicated that the interactions are insignificant and can be neglected in all the systems considered, irrespective of the concentration of NaCl in aqueous solution. The values of *A*′ obtained changed in the range from almost −10^−2^ to 10^−3^.

**Fig. 3:**
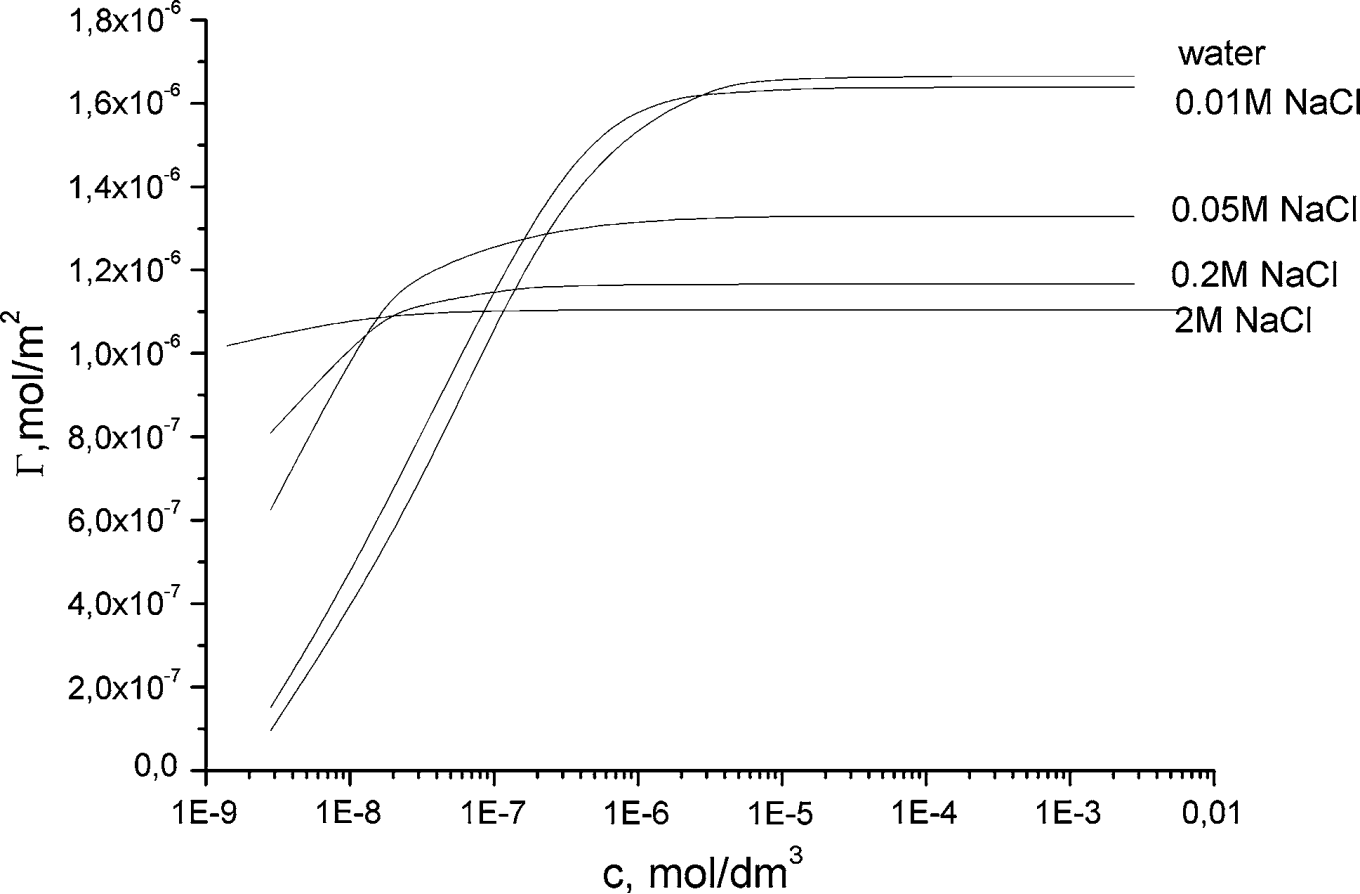
Surface excess isotherms for aqueous and salt solutions of cocobetaine

The efficiency of adsorption of cocobetaine in the various aqueous solutions is clearly different, too. This can be concluded from studies on surface excess isotherms shown in Fig. 3 which demonstrated higher efficiency of adsorption of cocobetaine molecules in water as diluents. In all cases, the surface excess isotherms increases monotonically to a constant value, characterized by the saturation of the phase boundary. Presentation of the adsorption isotherm in a semi-logarithmic system allows showing distinct differences between the various bulk concentrations of cocobetaine in various aqueous solutions, at which the saturation of the air/aqueous solution is observed. As can be seen the cocobetaine molecules in salts solution (0.05, 0.2 and 2 M) saturate phase boundaries at a much higher concentration compared to the water or 0.01 M NaCl solution.

Another important attribute of surfactants is the ability to wet out solid surfaces. Wetting of solid surfaces by liquids depends on both the surface and liquid [32].

The wetting properties are the ability to test product to dissolve in water, to lower the surface tension between liquid and solid, and to remove air from liquids and solids by aqueous solutions [33, 34]. One way to characterize the wetting of solid surfaces by a liquid is to measure the contact angle [35].

**Fig. 4:**
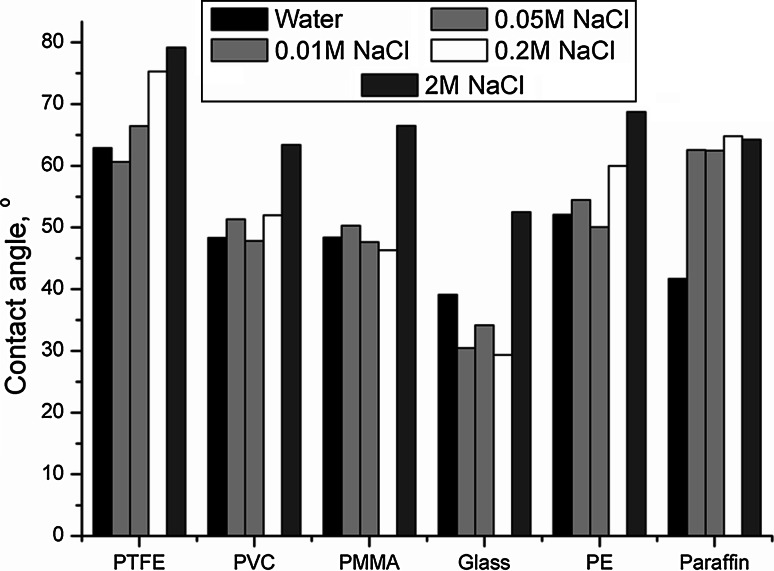
Contact angles of 0.1 % cocobetaine’s solutions for different surfaces

Solid surfaces with high surface energy are completely wetted by the liquids with high surface tension, respectively. As it can be seen in Fig. 4 wetting properties depend on solution of cocobetaine not for all surfaces examined. The presence of salt in the solutions effects only on wetting properties of Teflon, polyethylene and paraffin surfaces. Other examined surfaces were wetted by the aqueous and salt solutions almost to the same extent.

**Fig. 5:**
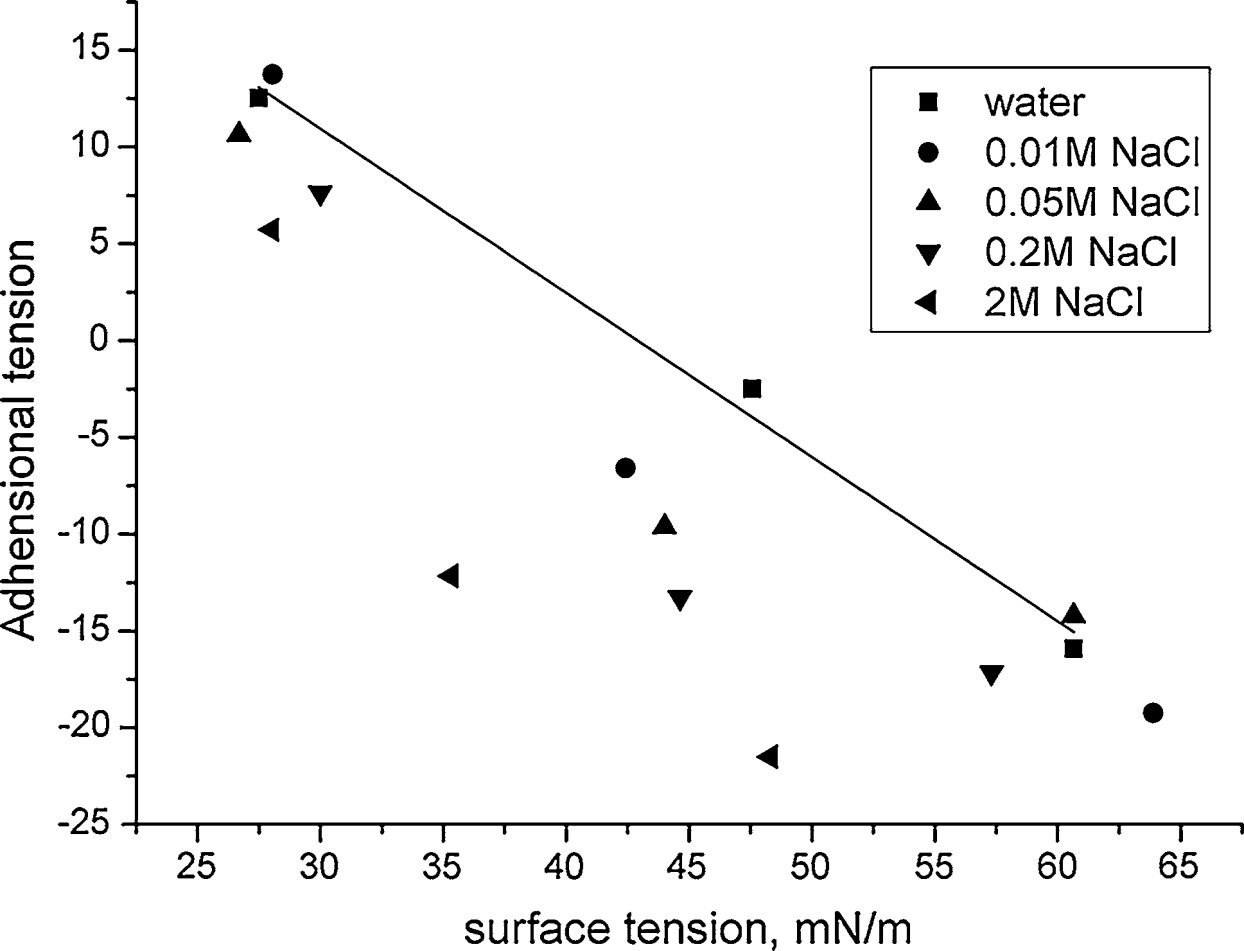
Dependence between the adhesional and surface tension of cocobetaine in different solutions

Bargeman and van Voorst Vader [36] found that there is a linear relationship between the adhesional tension (*γ* × cos(Θ)) and surface tension (*γ*) of aqueous solutions of surfactants:

$$\gamma \times { \cos }(\Theta ) = a \times \gamma + b,$$

where *a* and *b* are constants. They have also proposed that for nonpolar solids, such as a Teflon and surfactant system, the value of constant *a* is −1. The results of adhesional tension *vs* surface tension of aqueous solutions of cocobetaine for Teflon plate are presented in Fig. 4. The data shown in Fig. 5 confirms the conclusion of Bargeman and van Voorst Vader [36] that there is a linear relationship between these parameters. When all the lines have been drawn the figure was unreadable, therefore only the water system is shown. Regardless of the solvent used values of slope ranged are between −0.74 and −1.2. However, in contrast the a constant was positive in the case of contact angle measurements for the series of zwitterionic surfactants, alcohol hexadecyl polyoxyethylene glycidyl ether glycine betaines on the surface of the quartz plates (polar solids) [37].

**Fig. 6:**
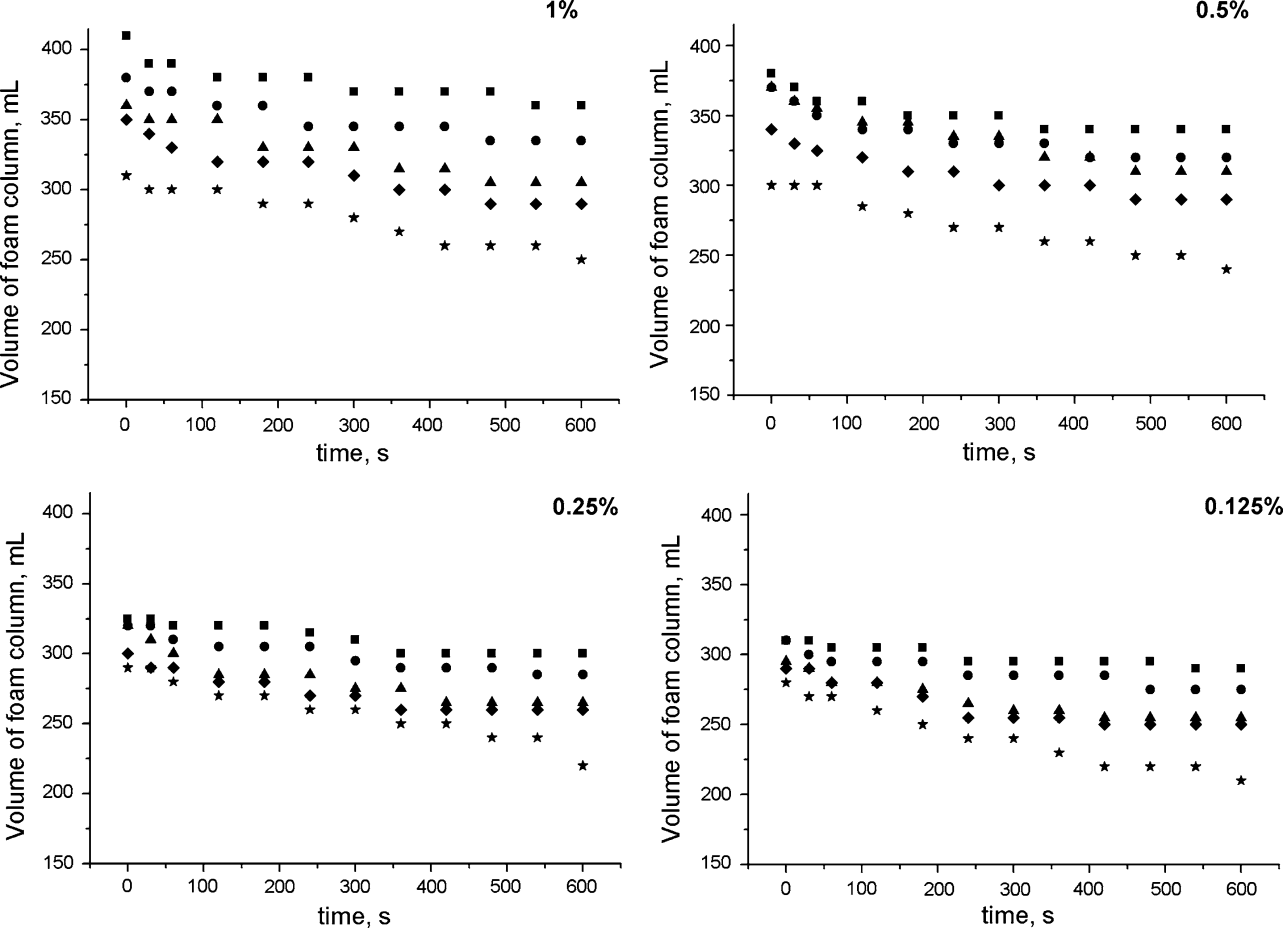
Volume of foam for cocobetaine solutions, *closed* *square* water, *closed* *circle* 0.01 M NaCl, *closed* *triangle* 0.05 M NaCl, *closed* *diamond* 0.2 M NaCl, *closed* *star* 2 M NaCl

Foaming ability is a typical property of surfactant solutions. The ability to produce foam is of great importance in cosmetics and household products. In the process of cleaning and washing the important role played by the quantity and durability of the produced foam [35]. The main factor determining the stability of the foam is the liquid drainage rate. The comparison of volume of foam aqueous and salt solutions of cocobetaine shows (Fig. 6) that the volume of foam for aqueous solutions is much higher than for appropriate salt ones. Thus, as well as surface properties, the ability of the foaming surfactant solution is better for water in comparison to the solution with addition of sodium chloride. The graph also shows that height of foam column is much lower for salt solutions then for aqueous solutions regardless of the time of measurement. Comparing the foaming capacity of aqueous solutions cocobetaine with aqueous solutions of soap, cocobetaine solutions show a larger volume of foam in the column. 2 % Soap solutions exhibit 290 cm^3^ of foam. This is much lower amount of foam than for the same solutions of cocobetaine [38].

Stability of foam was assessed by calculating the ratio of the initial foam volume to its value after 5 and 10 min. Main indicators applied to estimate the stability of the foam are: h5/h1index, h10/h1 index and constant speed of falling foam (k) [35]. Table 2 presents indexes estimated for several concentrations of cocobetaine aqueous and salt solutions. It can be noted that both indexes decreases with increasing concentration of salt. It can be also seen that the reduction of the foam in time is rapid for 2 M salt solutions of cocobetaine. The foam created for this solution disappears much faster than for aqueous solutions.

**Table 2:** Stability of foam indicators for cocobetaine solutions

	Aqueous solution		0.01 M salt solution		0.05 M salt solution		0.2 M salt solution		2 M salt solution	
Concentration of cocobetaine	h5/h1 index	h10/h1 index	h5/h1 index	h10/h1 index	h5/h1 index	h10/h1 index	h5/h1 index	h10/h1 index	h5/h1 index	h10/h1 index
0.125	0.97	0.95	0.97	0.93	0.93	0.91	0.91	0.89	0.89	0.78
0.25	0.97	0.94	0.95	0.92	0.93	0.88	0.93	0.90	0.93	0.79
0.5	0.97	0.94	0.94	0.91	0.94	0.87	0.92	0.89	0.90	0.80
1	0.95	0.92	0.93	0.91	0.94	0.87	0.94	0.88	0.93	0.83

## Conclusions

Based on the research and analysis carried out, it can be concluded that properties of cocamidopropyl betaine depend on the concentration of salt in the surfactant solutions. Experimental data showed that cocobetaine exhibits good surface properties and can be used as a substance which reduce surface tension even at low initial concentrations. The presence of salt in the solutions lessens their ability to reduce surface tension, decreases the CMC and increases the adsorption parameters and structure of the adsorbed monolayer. The experimental data showed that salt solutions of cocobetaine create a much smaller column of foam than aqueous solutions.

## List of symbols

*a*: Constant in adhesional tension equilibrium
*b*: Constant in adhesional tension equilibrium
*c*: Concentration (mol/dm^3^)
*A*′: Adsorption coefficient of the Frumkin isotherm
*A*_F_: Adsorption coefficient of the Frumkin isotherm (mol/dm^3^)
*A*_sz_: Adsorption coefficient of the Szyszkowski isotherm (mol/dm^3^)
*A*_min_: Minimum molecular area occupied by a statistical molecule at the saturated interface (nm^2^)
*B*_F_: Adsorption coefficient of the Frumkin isotherm
*B*_*sz*_: Adsorption coefficient of the Szyszkowski isotherm
CMC: Critical micelle concentration (mol/dm^3^)
*γ*_0_: Interfacial tension for concentration *c* = 0 (mN/m)
Δ*G*_ads_: Gibbs free energy of adsorption (kJ/mol)
Γ^∞^: Surface excess at the saturated interface (mol/m^2^)
Θ: Contact angle (°)
